# Distraction test of the posterior superior iliac spine (PSIS) in the diagnosis of sacroiliac joint arthropathy

**DOI:** 10.1186/1471-2482-13-52

**Published:** 2013-10-31

**Authors:** Clément M L Werner, Armando Hoch, Lucienne Gautier, Matthias A König, Hans-Peter Simmen, Georg Osterhoff

**Affiliations:** 1 Division of Trauma Surgery, University Hospital Zurich, Raemistrasse 100, Zurich 8091, Switzerland

**Keywords:** Sacroiliac joint pain, Provocation test, Joint infiltration, Diagnostic value

## Abstract

**Background:**

The sacroiliac joint (SIJ) is a frequently underestimated cause of lower back (LBP). A simple clinical test of sufficient validity would be desirable. The aim of this study was to evaluate the diagnostic value of a new PSIS distraction test for the clinical detection of SIJ arthropathy and to compare it to several commonly used clinical tests.

**Methods:**

Consecutive patients, where a SIJ pathology had been confirmed by an SIJ infiltration were enrolled (case group, 61 SIJs in 46 patients). Before infiltration, patients were tested for pain with PSIS distraction by a punctual force on the PSIS in medial-to-lateral direction (PSIS distraction test), pain with pelvic compression, pelvic distraction, Gaenslen test, Thigh Thrust, and Faber (or Patrick’s) test. In addition, these clinical tests were applied to both SIJs of a population of individuals without history of LBP (control group, 64 SIJs in 32 patients).

**Results:**

Within the investigated cohort, the PSIS distraction test showed a sensitivity of 100% and a specificity of 89% for SIJ pathology. The accuracy of the test was 94%, the positive predictive value (PPV) was 90% and the negative predictive value (NPV) was 100%. Pelvic compression, pelvic distraction, Gaenslen test, Thigh Thrust, and Faber test were associated with a good specificity (> 90%) but a poor sensitivity (< 35%).

**Conclusions:**

Within our population of patients with confirmed SIJ arthropathy the PSIS distraction test was found to be of high sensitivity, specificity and accuracy. In contrast, common clinical tests showed a poor sensitivity. The PSIS distraction test seems to be an easy-to-perform and clinically valuable test for SIJ arthropathy.

## Background

Low back pain (LBP) is a frequent and economically relevant disease with a cumulative life incidence of 70% [1]. It is one of the main causatives for long-lasting disability [2] and it is the most common cause of invalidity in the population with an age < 45 years [1]. The sacroiliac joint (SIJ) is a frequently underestimated cause of lower back and gluteal pain [3,4]. Some authors suggest that LBP is associated with or generated by the SIJ in 13% to 30% [3-5]. SIJ complaints usually occur after trauma, when practising certain types of sport, during pregnancy or after vaginal delivery, and after lumbar fusion surgery.

The complex anatomy of the SIJ as part of the pelvic ring and the variate aetiology of LBP often hinders finding a clinical diagnosis. Numerous clinical provocation tests have been described so far. However, their diagnostic validity – even when used in combination - is poor [5-9].

The gold standard for the diagnosis of SIJ-generated pain is the invasive technique of SIJ infiltration with local anaesthetics [5,6,8,10,11]. A decrease of complaints after infiltration indicates a pathology in the SIJ [12].

It would be desirable, however, to have a simple clinical test of sufficient validity to minimize the number of invasive diagnostic procedures. Fortin and Falco [13] first described the area around the posterior superior iliac spine (PSIS) to be the region of maximum patient-reported pain in patients with SIJ arthropathy. We developed a new clinical test where this knowledge is combined with provocation of SIJ movement, the PSIS distraction test. In our daily experience, this test was associated with a high rate of agreement with the presence of SIJ pathology.

Thus, it was the aim of this study to evaluate the diagnostic value of the PSIS distraction test for the clinical detection of SIJ arthropathy and to compare it to several commonly used clinical tests.

## Methods

We performed a retrospective study in a case–control design at a specialized centre for spine and pelvic surgery in Switzerland. The study was conducted in compliance with the Helsinki Declaration and was approved by the institutional review board (Kantonale Ethik kommission Zürich, Ref. No. 2011–0390). All patients and volunteers gave informed consent to the infiltration procedure and to participate in this study, respectively.

### Patients

**Figure 1:**
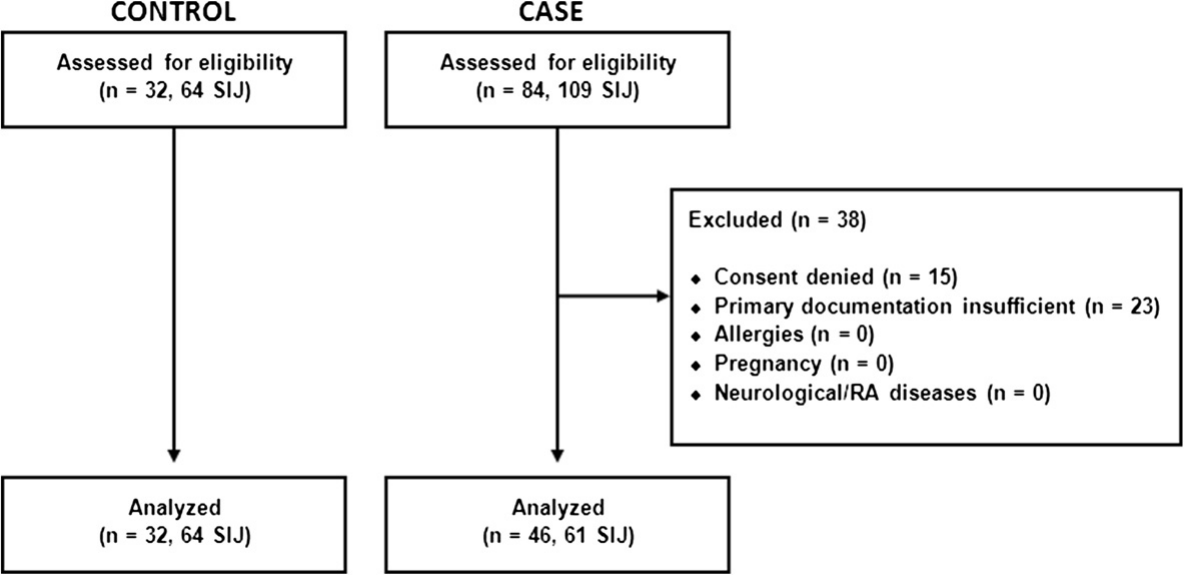
**Flow chart.** Flow chart showing inclusion and exclusion of patients**.** Forty-eight patients with 109 symptomatic SIJ were enrolled and after exclusion of 38 patients, 46 patients with 61 symptomatic SIJ were analyzed. For the control group, 32 patients with 64 asymptomatic SIJ were enrolled and analyzed.

Between June 2012 and February 2013 all consecutive patients aged ≥ 18 years with LBP where other sources of LBP (that is spondylarthropathies, radiculopathies, traumata, malignancies, and pathologies of the hip joint) had been excluded and a SIJ pathology had been confirmed by an SIJ infiltration, were enrolled for this study (case group, 61 SIJs in 46 patients, age 60 ± 13 years). If a patient had complaints in both SIJs, each joint was analyzed separately. Patients with pre-existing neurological or rheumatoid diseases, patients allergic to one of the substances used for SIJ-infiltration, those with severe dementia as well as pregnant patients were excluded (Figure 1).

In addition, the clinical tests described below were applied to both SIJs of a population of volunteers without any history of LBP or pelvic pain (control group, 64 SIJs in 32 patients, age 40 ± 15 years).

### Sacro-iliac joint infiltration

Within a period of maximum two weeks after the primary examination described below, infiltration of the SIJ was performed in an operation room with the patient in prone position. Under fluoroscopic guidance, a 19.5 Gauge Chiba needle was introduced into the painful SIJ. After the correct needle position was confirmed by the patient’s recognition of characteristic pain or – in cases of doubt - by instillation of a contrast agent, local anesthetics (5–8 ml Mepivacain 2%) and a steroid (40 mg Kenacort) were injected. All interventions were made by one of four investigators (CW, LG, MK, GO) in a non-blinded manner.

Directly before and after the infiltration, patients were asked to write down the level of pain according to the Visual Analogue Scale (VAS) in a pain log (Additional file 1). A decrease of VAS by ≥ 50% 15 to 30 minutes after infiltration was considered to indicate an intraarticular pathology of the SIJ.

### Clinical tests

7During routine examination before infiltration, all patients were tested for pain with PSIS distraction, pain with pelvic compression, pelvic distraction, Gaenslen test, Thigh Thrust, and Faber (or Patrick’s) test [5,6,8,10,11].

**Figure 2:**
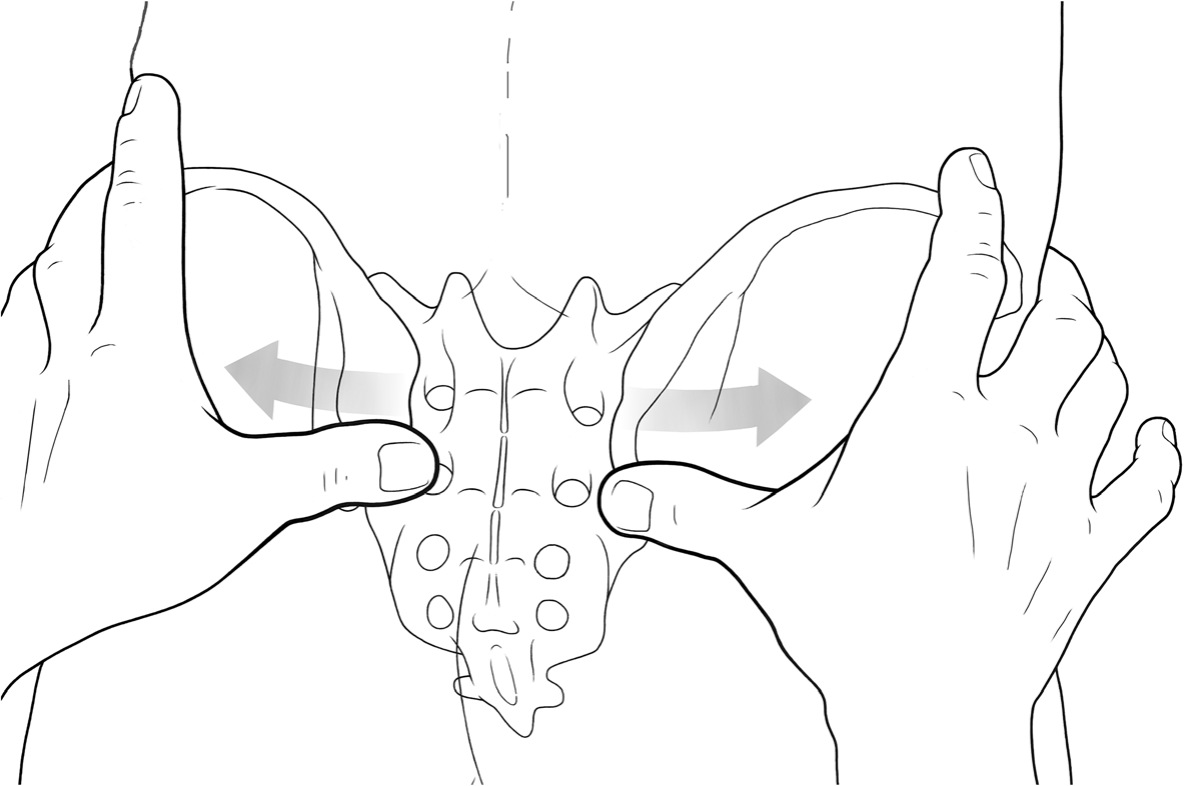
**PSIS distraction test.** A punctual force is applied on the PSIS in medial-to-lateral direction with the patient either standing or lying prone. The test is positive if the patient reports production of new or aggravation of pain.

For the new PSIS distraction test, the patients were asked whether they felt production of new or aggravation of pain when a punctual force was applied on the PSIS in medial-to-lateral direction with the patient either standing or lying prone (Figure 2). The test was considered positive if it reproduced the patient’s symptoms.

These tests were performed by one of four examiners (CLW, LG, MAK, GO). As this routine examination was performed before infiltration, examiners were blinded for the result of the SIJ infiltration of the patients with SIJ pathology in the case group. The investigator testing the non-symptomatic volunteers in the control group (GO) was not blinded.

### Statistical analysis

Age is expressed as mean ± SD. Diagnostic test evaluation was done using SPSS for Windows 21.0 (SPSS, Chicago, Illinois, USA).

## Results

A positive *PSIS distraction test*, defined by production of new or aggravation of pain when a punctual force was applied on the PSIS in medial-to-lateral direction was witnessed in all of the 61 SIJs (100%) where a SIJ pathology had been confirmed by infiltration. In the control group, the PSIS distraction test was positive in only seven of the 64 SIJs (11%) without any history of LBP or previous pelvic pain.

Within the investigated cohort, which did include only patients with confirmed SIJ pathology as the case group, the PSIS distraction test showed a sensitivity of 100% and a specificity of 89% for SIJ pathology. The accuracy of the test was 94%, the positive predictive value (PPV) was 90% and the negative predictive value (NPV) was 100%.

The diagnostic odds ratio of the PSIS distraction test was infinite.

Pain with *pelvic compression* was observed in 16 of the 61 SIJs (26%) with a SIJ pathology and in none of the 64 SIJs (0%) in the healthy control group (sensitivity 26%, specificity 100%, PPV 100%, NPV 59%).

Pain with *pelvic distraction* was observed in 14 of the 61 SIJs (23%) with a SIJ pathology and in one of the 64 SIJs (2%) in the control group (sensitivity 23%, specificity 98%, PPV 93%, NPV 57%).

A positive *Gaenslen test* and a positive *Thigh Thrust* were each found in 19 of the 61 SIJs (31%) with a SIJ pathology and in four of the 64 SIJs (6%) in the control group (each with a sensitivity of 31%, a specificity of 94%, a PPV of 83%, and a NPV of 59%).

A positive *Faber test* was witnessed in 21 of the 61 SIJs (34%) with a SIJ pathology and in five of the 64 SIJs (8%) in the control group (sensitivity 34%, specificity 92%, PPV 81%, NPV 60%).

## Discussion

The aim of this study was to evaluate the diagnostic value of a new clinical test for SIJ arthropathy, the PSIS distraction test, and to compare it to commonly used clinical tests.

Within our population of patients with confirmed SIJ arthropathy, the PSIS distraction test was found to be of high sensitivity, specificity and therefore a very good accuracy.

While other common clinical tests as pelvic compression, pelvic distraction, Gaenslen test, Thigh Thrust, and Faber test also showed a good specificity, their sensitivity was poor (< 35%).

This is in accordance with previously published data, even though the range of reported values for sensitivity and specificity is broad [6-9] and some of these studies lack control groups [8]. In a large systematic review, Szadek et al. analysed SIJ provocation tests (compression, distraction, thigh thrust, Gaenslen’s test, and Patrick’s sign/Faber test) and observed significant heterogeneity and inconsistency. They assumed the use of different thresholds for a positive reference standard of sufficient pain relief to be partly responsible for this [7].

Some authors reported an association [9,13,14] between patient-reported pain in the area of the posterior superior iliac spine (PSIS) and SIJ-generated pain. Still, a clinical test applying a force on the PSIS combining the knowledge of patient-reported pain with movement of exclusively the SIJ has not been described, so far.

The presence of pain-transducing nerve fibers in the SIJ and its adjacent ligaments is known [12,15]. In this context is important to know that the innervation of the sacroiliac joint is almost exclusively derived from the sacral dorsal rami [16,17]. The immediate anatomical vicinity of these neural structures and the PSIS could be an explanation for the good diagnostic value of the PSIS distraction test we observed. Similar to the supra- and infraorbital exits of the trigeminal nerve, the PSIS might serve as a pressure point to test the sensitivity of the sacral dorsal rami innervating the SIJ. However, a distinctive mapping of the nociceptive areas in the SIJ to strengthen this hypothesis has not been described in the literature.

Even though our study design was useful to differentiate between cases and the controls, the results need caution to some limitations. The clinical value of the PSIS distraction test was investigated on base of a case–control design. Since the case group consisted of patients with a SIJ arthropathy confirmed by an infiltration, this might especially influence the sensitivity values.

The commonly used SIJ provocative test are known to be subject to considerable inter- and intraobserver variabilities [7]. Information on this issue for the PSIS distraction test is not given by our study. We are convinced, however, that the simplicity of the PSIS distraction test procedure makes a high inter- and intraobserver variability unlikely.

The examiners were not blinded for the non-symptomatic volunteers in the control group. This might be a potential source of bias and in this case partially explain the good specificity. In contrast, examiners were not able to predict the results of the SIJ infiltration in the group of patients with SIJ pathology.

Further validation by prospective, blinded multi-investigator trials with a larger cohort is needed to confirm this assumption.

We did not differentiate on symptom duration before treatment. Pain mechanisms are different in acute, sub-acute or chronic pain and hyperalgesia in patients with a chronic pain syndrome is a possible confounding factor.

As many others [5,8,13,14], our study used SIJ infiltration under fluoroscopic monitoring as reference test. The topography of the sacroiliac joint space is variable as well as irregular and even with the use of radiopaque agent to prove the intraarticular position of the needle, there is a notable probability of an extraarticular infiltration leading to a false-negative test result. Thus, it has been not definitely proven if the tenderness of the PSIS is really caused by an intraarticular pathology or rather a periarticular (i.e. ligamenteous) problem. CT-guided infiltration may be used in future studies to avoid this vagueness.

Therefore, while the PSIS distraction test is not entirely validated trough this study, strong evidence on its clinical value is given. In contrast to the commonly described provocative tests, it can be performed with the patient standing and is easy to perform during clinical routine. Thereby, it provides a quick and robust decision guidance towards the need for more invasive diagnostics as SIJ infiltration.

## Conclusion

In conclusion, the PSIS distraction test was found to be of high sensitivity, specificity and accuracy within our population of patients with confirmed SIJ arthropathy. In contrast, common clinical tests as pelvic compression, pelvic distraction, Gaenslen test, Thigh Thrust, and Faber test showed a good specificity but their sensitivity was poor.

Therefore, the PSIS distraction test seems to be an easy-to-perform and clinically valuable test for SIJ arthropathy.

## Competing interests

The authors declare that they have no competing interests.

## Authors’ contributions

CW had the idea of the PSIS test, participated in designing the study, analysed the data and drafted the manuscript. AH, LG and MK participated in data acquisition and revised the manuscript. HPS was involved supervised the study and and revised the manuscript. GO participated in designing the study, the analysis of the data and in drafting the manuscript. All authors read and approve the final manuscript.

## Pre-publication history

The pre-publication history for this paper can be accessed here:

http://www.biomedcentral.com/1471-2482/13/52/prepub

## Supplementary Material

Additional file 1 Pain log for the documentation of VAS after SIJ infiltration. Click here for file

## References

[B1] Andersson GB Epidemiological features of chronic low-back pain Lancet 1999 354 9178 581 585 10.1016/S0140-6736(99)01312-4 10470716

[B2] Collaborators USBoD The state of US health, 1990–2010: burden of diseases, injuries, and risk factors JAMA 2013 310 6 591 608 10.1001/jama.2013.13805 23842577 PMC5436627

[B3] Daum WJ The sacroiliac joint: an underappreciated pain generator Am J Orthop (Belle Mead NJ) 1995 24 6 475 478 7670870

[B4] DonTigny RL Anterior dysfunction of the sacroiliac joint as a major factor in the etiology of idiopathic low back pain syndrome Phys Ther 1990 70 4 250 265 discussion 262–255 2138334 10.1093/ptj/70.4.250

[B5] Schwarzer AC Aprill CN Bogduk N The sacroiliac joint in chronic low back pain Spine (Phila Pa 1976) 1995 20 1 31 37 10.1097/00007632-199501000-00007 7709277

[B6] Foley BS Buschbacher RM Sacroiliac joint pain: anatomy, biomechanics, diagnosis, and treatment Am J Phys Med Rehabil 2006 85 12 997 1006 10.1097/01.phm.0000247633.68694.c1 17117004

[B7] Szadek KM van der Wurff P van Tulder MW Zuurmond WW Perez RS Diagnostic validity of criteria for sacroiliac joint pain: a systematic review J Pain 2009 10 4 354 368 10.1016/j.jpain.2008.09.014 19101212

[B8] Slipman CW Sterenfeld EB Chou LH Herzog R Vresilovic E The predictive value of provocative sacroiliac joint stress maneuvers in the diagnosis of sacroiliac joint syndrome Arch Phys Med Rehabil 1998 79 3 288 292 10.1016/S0003-9993(98)90008-9 9523780

[B9] Dreyfuss P Michaelsen M Pauza K McLarty J Bogduk N The value of medical history and physical examination in diagnosing sacroiliac joint pain Spine (Phila Pa 1976) 1996 21 22 2594 2602 10.1097/00007632-199611150-00009 8961447

[B10] Broadhurst NA Bond MJ Pain provocation tests for the assessment of sacroiliac joint dysfunction J Spinal Disord 1998 11 4 341 345 9726305

[B11] Stone JA Bartynski WS Treatment of facet and sacroiliac joint arthropathy: steroid injections and radiofrequency ablation Tech Vasc Interv Radiol 2009 12 1 22 32 10.1053/j.tvir.2009.06.009 19769904

[B12] Szadek KM Hoogland PV Zuurmond WW De Lange JJ Perez RS Possible nociceptive structures in the sacroiliac joint cartilage: an immunohistochemical study Clin Anat 2010 23 2 192 198 20014392 10.1002/ca.20908

[B13] Fortin JD Falco FJ The Fortin finger test: an indicator of sacroiliac pain Am J Orthop (Belle Mead NJ) 1997 26 7 477 480 9247654

[B14] Murakami E Aizawa T Noguchi K Kanno H Okuno H Uozumi H Diagram specific to sacroiliac joint pain site indicated by one-finger test J Orthop Sci 2008 13 6 492 497 10.1007/s00776-008-1280-0 19089535

[B15] Vilensky JA O’Connor BL Fortin JD Merkel GJ Jimenez AM Scofield BA Kleiner JB Histologic analysis of neural elements in the human sacroiliac joint Spine (Phila Pa 1976) 2002 27 11 1202 1207 10.1097/00007632-200206010-00012 12045518

[B16] Fortin JD Kissling RO O’Connor BL Vilensky JA Sacroiliac joint innervation and pain Am J Orthop (Belle Mead NJ) 1999 28 12 687 690 10614759

[B17] Grob KR Neuhuber WL Kissling RO Die Innervation des Sacroiliacalgelenkes beim Menschen Z Rheumatol 1995 54 117 122 7793158

