# Supplementary material for: Distraction test of the posterior superior iliac spine (PSIS) in the diagnosis of sacroiliac joint arthropathy
Source: BMC Surg. 2013 Oct 31;13:52. doi: 10.1186/1471-2482-13-52 (PMC3827936; doi:10.1186/1471-2482-13-52)
Supplement: Additional file 1 — Pain log for the documentation of VAS after SIJ infiltration. [file 1471-2482-13-52-S1.pdf]

UniversitätsSpital Zürich

Klinik für Unfallchirurgie

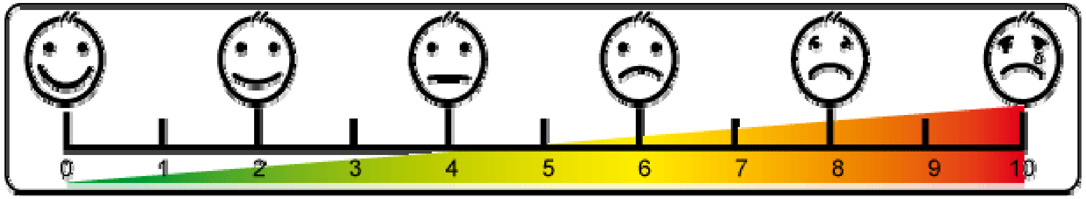

Patientenkleber

Patientenprotokoll Schmerzintensität	Infiltration	Typ	Ort	Seite	Datum

VAS	Vortag	nach 1 Stunde	nach 6 Stunden	1. Tag Morgen	Abend	2. Tag Morgen	Abend	3.Tag	4.Tag	7. Tag
**10**										
**9**										
**8**										
**7**										
**6**										
**5**										
**4**										
**3**										
**2**										
**1**										
**0**										
**Bemerkungen**										
